# Th17 lymphocytes and alternatively activated monocytes are upregulated in clinical sepsis

**DOI:** 10.1186/cc10169

**Published:** 2011-06-22

**Authors:** R Salomão, M Brunialti, M Santos, O Rigato, F Machado, E Silva

**Affiliations:** 1 Escola Paulista de Medicina, UNIFESP, São Paulo - SP, Brazil

## Introduction

Sepsis is a systemic inflammatory response triggered by infection. Inflammatory response is modulated during sepsis and upregulation and downregulation of cellular activity is observed, depending on the cells and functions evaluated. Nevertheless, the interaction of innate and adaptative immune responses has been little studied in clinical sepsis.

## Objective

The aims of this study were to evaluate the presence of TCD4 lymphocytes Th1, Th17, regulatory (Treg) and alternatively activated monocytes in septic patients and their association with prognosis.

## Methods

Septic patients were enrolled at admission (D0, *n *= 67) and after 7 days of therapy (D7, *n *= 33). Thirty-two healthy volunteers matched for age and gender were included as controls. PBMC were obtained by the Ficoll gradient method. Th1 and Th17 lymphocytes were identified by the intracellular detection of IFNγ and IL-17, respectively, and Treg cells were identified by Foxp3^+^CD127^- ^or CD25^+^CD127^- ^expression. Monocytes were evaluated for CD206 and CD163 expression.

## Results

Spontaneous production of IFNγ and IL-17A was increased in TCD4 cells of septic patients when compared with healthy volunteers. After PMA/Io stimulation, the percentage of TCD4 lymphocytes producing IFNγ was lower and IL-17 was higher in septic patients than in healthy volunteers. The results based on absolute TCD4^+ ^lymphocyte counting showed a lower proportion of Th1 cells and double the proportion of Th17 cells in septic patients compared with healthy volunteers while the proportion of Treg remained unchanged. In follow-up samples, a higher percentage of IFNγ and a lower percentage of IL-17 producing cells were observed compared with D0 samples. A higher percentage of spontaneously producing IFNγ was found in D7 compared with D0 samples from patients who died and a decreased percentage of PMA/Io-induced IL-17 producing cells between patients' samples of follow-up (D7) compared with admission samples was found in survivors. Septic patients showed a markedly increased proportion of alternatively activated monocytes, which was sustained in both patients' samples.

## Conclusion

We found a decreased proportion of Th1 and increased proportion of Th17 in septic patients, and an impressive increase in the percentage of monocytes expressing CD206 and CD163, indicating differentiation towards wound healing and regulatory or inhibitory monocytes, which may underscore the previous studies showing a reprogramming of monocytes' function in sepsis.

